# Primary care patients with cardiovascular disease eligible for nurse‐led internet‐based cognitive behavioural therapy for insomnia: Characteristics and motives for participation

**DOI:** 10.1002/nop2.1717

**Published:** 2023-03-17

**Authors:** Sandra Öberg, Linda Johansson, Mattias Georgsson, Jonas Sandberg, Anders Broström

**Affiliations:** ^1^ Department of Nursing Science, School of Health and Welfare Jönköping University Jönköping Sweden; ^2^ School of Health and Welfare, Aging Research Network‐Jönköping, Institute of Gerontology Jönköping University Jönköping Sweden; ^3^ Department of Nursing Science Sophiahemmet University Stockholm Sweden; ^4^ Department of Clinical Neurophysiology Linköping University Hospital Linköping Sweden

**Keywords:** cardiac disease, characteristics, cognitive behavioural therapy, insomnia, internet‐based intervention, mixed method design, nurse‐led, sleep problems

## Abstract

**Aim:**

To describe demographic, physical and psychological characteristics associated with insomnia in patients with cardiovascular disease (CVD) participating in nurse‐led Internet‐based cognitive behavioural therapy for insomnia (I‐CBTI), and their motives and expectations regarding participation in I‐CBTI.

**Design:**

A mixed method design was applied, including primary care patients with angina pectoris, myocardial infarction, heart failure, atrial fibrillation and atrial flutter or arrhythmia in southern Sweden.

**Methods:**

Data on demographics, insomnia severity and physical and psychological characteristics were collected through self‐rated validated questionnaires (*n* = 126). Motives and expectations were collected through interviews (*n* = 19) and analysed using the ‘personas’ model.

**Results:**

Physical symptoms and psychological characteristics were associated with insomnia. Three personas were identified: the pragmatist (a curious and optimistic persona), the philosopher (a problem‐solving persona) and the philanthropist (an altruistic persona). Expectations were positive among the three personas, but comorbid conditions reduced the perceived ability to make necessary behavioural changes.

## 1 INTRODUCTION

In the general adult population, 10%–50% report complaints about insufficient sleep (Bhaskar et al., 2016). An important aspect for nurses to consider is that sleep complaints, not only are common, but they are also often associated with several types of cardiovascular disease (CVD; Wang et al., 2016). For example, self‐reported short sleep duration (≤6 h) was in a large sample identified as a potential causal risk factor for five CVDs (i.e., arterial hypertension, pulmonary embolism, coronary artery disease, myocardial infarction and chronic ischaemic heart disease; Ai et al., 2021). Itani et al. (2017) found in a recent meta‐analysis short sleep to be significantly associated with hypertension, cardiovascular diseases, coronary heart diseases and mortality, with the latter also stated by Yin et al. (2017) to cover long sleep (>8 h). Others have found both short and long sleep to increase the risk of overall CVD mortality (Krittanawong et al., 2019), particularly among older men with cardiovascular morbidity (Brostrom et al., 2018). Moreover, Wang et al. (2022) found napping and poor sleep patterns to be associated with an increased CVD risk. On the contrary, Häusler et al. (2019) found napping to lower the risk of incident CVD events.

Sleep problems can manifest differently. Ten percent of sleep complaints among adults are caused by insomnia; a common condition, especially among primary care patients, which may be explained by comorbidities that lead the patients to consult health care services (Léger et al., 2010). Insomnia has a bidirectional association with CVD as well as a high comorbidity with psychiatric conditions (i.e., depression and anxiety). The diagnostic criteria for insomnia are based on having difficulties initiating or maintaining sleep or waking up earlier than desired, at least three times a week for a minimum of 3 months, and with at least one reported daytime impairment related to the sleep difficulty (Riemann et al., 2017). Looking specifically at CVD and insomnia comorbidities (Javaheri & Redline, 2017) found an increased risk of morbidity and mortality as well as poor quality of life. Even so, the prevalence of insomnia among patients with CVD has been sparsely studied, but the previous occurrence of 37% in acute coronary disease (Coryell et al., 2013) and 50% in heart failure (Redeker et al., 2010), together with more recent studies focusing associations between sleep problems, from a more general perspective and CVD (Ai et al., 2021) indicates sleep to be an important clinical topic with a great need to identify effective interventions.

## 2 BACKGROUND

Cognitive behavioural therapy (CBT) is the recommended treatment for insomnia according to recent guidelines (Riemann et al., 2017). Despite the proven positive long‐term effects of CBT, sleep medication is a commonly used treatment alternative (Grandner et al., 2016), although associated with several negative side effects (e.g. morning sedation, dependence and impaired balance; Buysse, 2013). One potential explanation for the high occurrence of sleep medication prescriptions might be the limited access to CBT therapists, in both hospital and primary care settings. However, internet‐based CBT for insomnia (I‐CBTI) may be one way to offer enhanced treatment access (Riemann et al., 2017). I‐CBTI has shown positive research outcomes for mental disorders (e.g. depression and anxiety) and in patients with different chronic diseases (e.g. pain, cancer and cardiovascular disease; Mehta et al., 2019). It has also been shown to be equally as effective as face‐to‐face CBT for insomnia (Blom et al., 2015). An I‐CBTI programme contains different components such as keeping a sleep diary, taking part in sleep hygiene education and psychoeducation on sleep behaviour changes and how to manage setbacks regarding sleep difficulties in the future (Zachariae et al., 2016). It is also suggested to be beneficial to develop behavioural interventions focusing on both the psychological comorbidities (i.e. insomnia) along with CVD factors simultaneously to enhance the overall well‐being of this patient group (Davidson et al., 2018). In a few studies, sleep improvement and decreased levels of anxiety, depression (Heenan et al., 2020) and fatigue (Redeker et al., 2015) have been the result when using tailored I‐CBTI interventions for patients with CVD. The tailoring in the mentioned studies meant including CVD‐related components in the treatment (e.g. taking cardiac medication, sleep consequences for the cardiac system, insomnia after surgery and the fear of cardiac‐related thoughts and events at night; Heenan et al., 2020; Redeker et al., 2015). Even so, I‐CBTI interventions for patients with CVD are rare (Nygardh et al., 2017) but are slowly becoming recognised as possible treatment options to improve sleep and quality of life (Siebmanns et al., 2021). However, the patient's perspective about engaging in a tailored intervention is important to investigate, since the individual's current motivation is related to the decision to carry out behavioural change (Schwarzer, 2008). Ferwerda et al. (2013) explored patients' perspectives on I‐CBT treatment for patients with chronic disease (i.e. rheumatoid arthritis and psoriasis). The finding showed that the participants described several possible advantages (Ferwerda et al., 2013). Aspects such as no travel time, no waiting time, being able to decide when to spend time on treatment and that it might be easier to share personal problems were mentioned. Most of the participants (80%) in the study reported being positive towards participating in an I‐CBT intervention (Ferwerda et al., 2013).

Recent interventional studies suggest that I‐CBTI treatment for patients with CVD has a positive effect on sleep outcomes (Javaheri et al., 2020) as well as improved mental well‐being (Redeker et al., 2015). However, I‐CBTI treatments tailored to patients with CVD are relatively unexplored and additional research is needed to further understand the elements that could influence adherence and eligibility for such treatment. The aims of the study were, therefore, to (a) describe the demographic, physical and psychological characteristics associated with insomnia in primary care patients with CVD interested in participating in I‐CBTI, and (b) to determine their expressed motives and expectations concerning participating in such an intervention.

## 3 METHOD

### 3.1 Study design and sample

**FIGURE 1:**
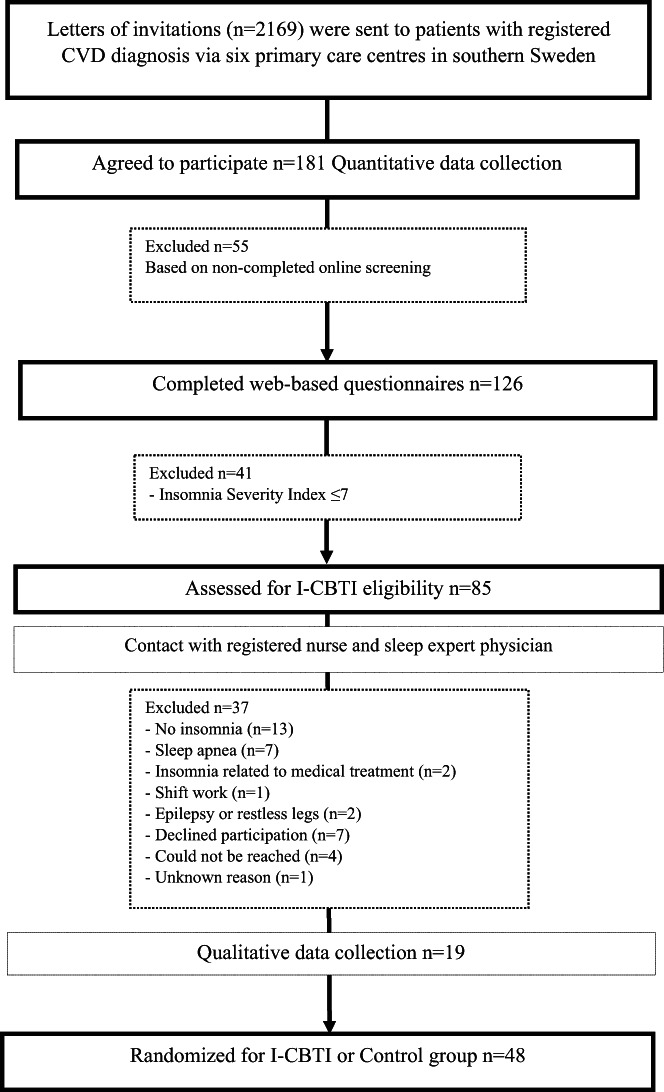
Flowchart of the inclusion process of patients with cardiovascular disease interested in participating in the internet‐based cognitive behavioural therapy for insomnia.

A mixed method design was adopted (Appendix S1), where the quantitative and qualitative data was collected, analysed and presented in a sequence and the results are integrated into the discussion section (Ivankova et al., 2006). The purpose of using a mixed method design was to explain the participants' sleep, physical and psychological health characteristics in relation to their motives and expectations to participate in a I‐CBTI. The participants were recruited from six primary care centres in southern Sweden with the intent to be part of a nurse‐led I‐CBTI study tailored to patients with CVD (Siebmanns et al., 2021). The current study has a cross‐sectional approach with web‐based questionnaires collected before the intervention to assess the participants' insomnia severity and their physical and psychological characteristics. This method was supported by semi‐structured interviews to reveal the participants' motives and expectations concerning participating in an I‐CBTI study (Figure 1). The inclusion criteria for the current study was to have a verified International classification of disease (ICD‐10), i.e., angina pectoris (I20.9, I20.9P), myocardial infarction (I25.2, I25.‐P), heart failure (I50.9, I50.‐), atrial fibrillation and atrial flutter (I48.9, I48.‐) and arrhythmia NOS (I49.9, I49) or the Classification of diseases and health problems 1997 (KSH97‐P). The inclusion criteria for the original nurse‐led I‐CBTI study were to have completed the web‐based questionnaire, to have self‐rated insomnia (ISI ≥8) and at least two registered visits to primary care in relation to a CVD diagnosis, and access to the internet, a computer or smartphone. Inclusion criteria for interview participation were to have self‐rated insomnia (ISI ≥8) and an assessed insomnia diagnose according to the ICDS‐3 criteria (Riemann et al., 2017) made by a physician specialised in sleep medicine. Exclusion criteria were the need for an interpreter to understand Swedish text and language, diagnosis of cognitive impairments, severe psychiatric and somatic disease, restless legs syndrome or untreated sleep apnoea, epilepsy, drug abuse or expected survival of less than 12 months. The inclusion process is described in Figure 1. Of 2169 study invitations, 181 patients agreed to participate in the intervention study. All participants gave their informed written consent to participate in the I‐CBTI study. The research project was approved by the Regional Ethical Council (Dnr 2015/258‐31, 2017/378‐32).

### 3.2 Data collection

A data collection flowchart is presented in Figure 1. The participants that agreed to participate were emailed a link to complete a web‐based questionnaire regarding demographics (i.e. age, gender, living status, type of CVD, other comorbidities, weight and height to get an automatically calculated BMI), number of medications, sleep situation (i.e. insomnia severity and efficiency as well as sleep medication use) and physical and psychological well‐being (i.e. somatic symptom severity, mental and physical quality of life, cardiac anxiety, adaptability and personality trait). Of the 181 patients, 126 completed the web‐based questionnaire, of which 85 noted self‐perceived insomnia on a validated insomnia‐specific instrument (i.e. the Insomnia Severity Index). These participants were contacted by the study nurse via telephone to assess a preliminary inclusion criteria approval. Those eligible were then scheduled to be examined by a sleep expert physician to determine clinical insomnia. Forty‐eight participants of those examined were assessed as eligible to be randomised for the I‐CBTI intervention.

Participants for interviews (*n* = 19) were consecutively selected among those who had completed the quantitative part and satisfied the following criteria: completed the web‐based questionnaire with self‐rated insomnia (ISI ≥8) and assessed insomnia diagnose according to the ICDS‐3 criteria (Riemann et al., 2017) made by a physician specialised in sleep medicine. The participants gave their written consent to be interviewed about their motives and expectations for participating in the I‐CBTI study. The interviews were completed prior to the I‐CBTI randomisation (Figure 1) and conducted face‐to‐face by the first author in a primary care setting or at the participant's home. The interviews were semi‐structured (mean length 40 min), to explore the aim of the study questions such as: ‘Can you describe what motivated you to participate in the intervention study?’ ‘What expectations do you have?’ followed by probing questions that were used to understand the patient's notions of the intervention programme and CBT as a treatment method.

### 3.3 Measurement

#### 3.3.1 Insomnia characteristics

The Insomnia Severity Index (ISI) includes seven items. It was used to measure the nature, severity and impact of insomnia severity during the previous month, and included questions on the ability to fall asleep and to stay asleep, or difficulties with early awakenings, sleep satisfaction, interference of the sleep situation with daily life, how the sleep situation was noticed by others and the distress caused by the sleep difficulties. A five‐point Likert scale was used for each item to rate the insomnia severity from no problem (0) to a very severe problem (4) and yielded a summary score from 0 to 28 (Morin et al., 2011). The insomnia severity is divided into four levels: no clinical insomnia (0–7), subthreshold insomnia (8–14), clinical insomnia of moderate severity (15–21) and severe clinical insomnia (22–28) (Sarsour et al., 2010). The ISI instrument is a valid and reliable instrument to detect insomnia (Morin et al., 2011). The Cronbach's α for ISI was 0.68. The Pittsburgh Sleep Quality Index (PSQI) is a validated and reliable instrument that measures the respondent's quality of sleep and the severity of specific sleep‐related problems (Mollayeva et al., 2016). The PSQI is summarised into seven component scores (Buysse et al., 1989). The Cronbach's α for PSQI item 5–9 was 0.769 (item 1–4 are open questions for time‐specific sleep routines). The one component score used in the current study represents the participant's sleep efficiency in percentages, divided into >85%, 75%–84%, 65%–74% and <65%.

#### 3.3.2 Physical characteristics

The Patient Health Questionnaire‐15 (PHQ‐15) includes 15 items and is a validated and reliable instrument to measure how bothered respondents are with somatic symptom severity (e.g. feeling tired, having little energy or trouble sleeping) over the two last weeks (Kroenke et al., 2002). The scores range from not bothered at all (0) to bother a lot (2). The level of somatic severity score was divided into minimal (0–4), low (5–9), medium (10–14), and high (15–30) (Kroenke et al., 2002). The Cronbach's α for PHQ‐15 was 0.78. The short form (12) health survey (SF‐12) includes 12 items and was used to measure the respondent's health‐related quality of life, involving their functional status, feelings and their valued health status. The SF‐12 includes physical health questions (physical functioning, role, physical or bodily pain) and mental health questions (mental health, role emotional and social functioning), which are reported with two summary scores—the physical component score (PCS) and the mental component score (MCS). A higher score on each summary indicates a better physical or mental quality of life (Ware Jr. et al., 1996) The SF‐12 is a valid and reliable instrument to measure respondents’ quality of life (Muller‐Nordhorn et al., 2004; Ware Jr. et al., 1996). The Cronbach's α for SF‐12 was 0.82. In the current study the health‐rated quality of life (EQ‐5D) visual analogue scale (EQ‐VAS) was used to capture a self‐rated health status from 0 (worst imaginable health state) to 100 (best imaginable health state), a higher score indicating better self‐perceived health (Rabin & De, 1990).

#### 3.3.3 Psychological characteristics

The Patient Health Questionnaire‐9 (PHQ‐9) includes nine items, and one extra item that measures the respondent's level of physical and social functionality (if reporting depressive symptoms). The PHQ‐9 is a validated and reliable instrument to measure the respondent's depressive symptoms with point scores from 0 to 3 (Kroenke et al., 2001). The nine items covered the following areas: interested in doing things, problems sleeping, feelings of depression, tiredness, appetite, self‐esteem, concentration level, level of physical movement and harming thoughts. The PHQ‐9 was used in the current study with summary score ranges from 0 to 27 divided into five levels of depression: minimal (0–4), mild (5–9), moderate (10–14), moderately severe (15–19) and severe (20–27) (Kroenke et al., 2001) The Cronbach's α for PHQ‐9 was 0.81. The Cardiac Anxiety Questionnaire (CAQ) is an 18‐item instrument that is a validated and reliable instrument to measure heart‐focused anxiety (Van Beek et al., 2012). The instrument includes a five‐point Likert scale ranging from never (0) to always (4) and identifies three subscales: heart‐focused fear, avoidance and attention (Van Beek et al., 2012). A higher score indicates greater heart‐focused anxiety (Eifert et al., 2000). The Cronbach's α for CAQ was 0.83. The seven‐item Mastery Scale (MSC) is a validated instrument (Eklund et al., 2012) that focuses on to what degree the respondents have control over their life situation using statements such as ‘I have little control over the things that happen to me’, with four categories of answers from 1 (totally agree) to 4 (strongly disagree), with 4 indicating the highest level of ‘mastery’. The summary score varies between 7 and 28, and a higher score indicates more feeling of control (Pearlin & Schooler, 1978). The Cronbach's α for MSC was 0.65. The 14‐item type D‐personality questionnaire (DS‐14) assesses personality traits in two categories: Negative affectivity (NA) and social inhibition (SI), and includes seven items each. NA refers to the experience of feeling dysphoria, anxiety and irritability and SI refers to discomfort in social interactions. Participants rate their personality traits on a five‐point Likert scale from false (0) to true (4) and a cut‐off scale ≥10 on both NA and SI is used to classify a type D‐personality. The DS‐14 is considered a valid and reliable instrument to detect negative emotions and/or emotional distress which is an important risk factor related to CVD development (Denollet, 2005). The Cronbach's α for DS‐14 was 0.82.

### 3.4 Data analysis

The statistical analysis was performed using IBM SPSS v26.0, performed at a 5% significance level. The analysis is based on the sample who completed the web‐based questionnaire (*n* = 126) and the two groups defined as eligible (*n* = 48; the participants who were randomised in the intervention) and excluded (*n* = 37; by own initiative or by not meeting the inclusion criteria). The demographics, sleep, physical and psychological characteristics total and group values were computed using descriptive statistic frequencies and crosstabs. Group comparison (eligible for randomisation or excluded) significance was analysed with an independent *t*‐test for parametric variables (age) whereas nominal variables (gender, living status, type of CVD, occurrence of high blood pressure, lipid disorder and personality trait) were analysed using a chi‐square test. Group comparison significance for educational level, BMI, number of medications, insomnia severity, quality of life, cardiac anxiety, adaptability, quality of life and somatic symptom severity were analysed using two independent sample tests (Mann–Whitney). Variables with an expected count of less than 5 were not analysed to prevent non‐valid results. Bivariate Correlation using Spearman's Rho was carried out among insomnia, physical and psychological outcomes.

The qualitative analysis was guided by the ‘personas’ model created by Cooper et al. (2007). The persona analysing process includes identifying the users' shared goals, behaviours and attitudes from interviews or observations and creating groups and descriptions representing the unique features of each group. The process involves identifying behavioural variables, mapping interview subjects to the behavioural variables, identifying behaviour patterns, synthesising the characteristics and relevant goals, checking for redundancy and fullness, expanding the description of attributes and behaviours and lastly designating the persona types (Cooper et al., 2007). The collected interview data from the 19 participants were considered sufficient to create persona types that would represent the participants' various clinical characteristics (i.e., cardiac conditions and insomnia problems), as well as motives and expectations regarding participating in the I‐CBTI study. The audiotaped interviews were transcribed, and the analysis process started by deciding on the behavioural themes according to the study objectives, i.e., the motives (why) and expectations (what) and to what degree those expectations were expressed (how) regarding participating in the I‐CBTI study. The interviews were then read several times to identify text segments describing each participant's motives and expectations and these were put in a table to further identify behavioural variables (Table 1). The identified variables of each participant's objective themes were listed and clustered into similar characteristic patterns. The identified characteristics were ‘sleep improvement‐oriented’, ‘sleep tool searcher‐oriented’ and ‘contributor‐oriented’, and the interviews were mapped in groups according to that specific pattern. This meant that each participant could only belong to one of the groups. The interviews in each group were then reread and key concepts were noted in an affinity diagram to synthesise the group characteristics in detail and determine each interview's belonging to the specific group (Table 2) which led to a few adjustments. A third‐person narrative description of each group was created, starting with a presentation of the characteristic, the problems they were facing, goals, motives to act and the attempts to reach those goals (Madsen & Nielsen, 2010). Lastly, a persona type was designated to represent each group's primary behavioural characteristics. These types were*: The pragmatist, the philosopher and the philanthropist*.

**TABLE 1:** Example of identified behavioural variables belonging to the ‘contributor oriented’ characteristics.

Themes	Text segments	Behavioural variables
Motives why	I have difficulty falling asleep and thought, I can get some advice and help in general if I cannot solve my problems myself. It is not certain that it will work, so in any case it can contribute to someone else being able to get the problem solved	Difficulty falling asleep Sleep advice Contribute to others
Expectations what	I thought maybe I could get some help, someone who had similar problems and solved it in some way	Sleep improvement
Expectations how	Well not so much, I realise it is like this, it is complex to sort things out. This is about the body and everything, so it can be difficult	Low expectations

**TABLE 2:** Example of key concepts from the affinity diagram that was used after the interview mapping to synthesise the characteristics of each group's motives and expectations for participating in the internet‐based cognitive behavioural therapy for insomnia.

Sleep improvement oriented	Sleep tool searcher oriented	Contributor oriented
Curious and optimistic Problem falling and staying asleep Sleeping problem for a long time Is ready to solve the sleeping problem Prefers non‐pharmacological treatment Has knowledge and interest in the treatment method Expects to receive help with the poor sleep	Problem‐solver Experiences daytime fatigue and trouble falling and staying asleep Wants to find a method to improve sleep Wants to change the sleep behaviour Wants specific sleep tool knowledge Expects new sleep knowledge Have worries that the comorbid conditions will hinder sleep improvement	Altruistic Problem falling and staying asleep Wants general sleep advice Wants to help others Has an interest in research Has low expectations that participation will solve the sleep problem related to the comorbid conditions

## 4 RESULTS

Demographics and sleep characteristics are shown in Table 3. The mean age among the 126 participants was 71 years (SD = 9.9) and there was more male (73%) than female participants, and the majority cohabited with another person (76%). University education (44%) was the most common degree, and myocardial infarction (46%) was the most frequent CVD diagnosis. Almost 70% of the participants reported insomnia symptoms (i.e. subthreshold to severe clinical insomnia) and 80% reported not having taken medicine to help with sleep in the last month. Minimal to mild depressive symptoms were reported by 90% of the participants, and 25% reported a type D personality.

**TABLE 3:** Demographics and sleep characteristics of patients with cardiovascular disease interested in participating in the internet‐based cognitive behavioural therapy for insomnia (I‐CBTI; *n* = 126).

Variables	Total	Eligible for ICBTI ISI 8–28	Excluded ISI 8–28	*p‐Value* ^b^	*Interview participant's*
*n*	126	48	37		
Age (M, SD)	71.07 (9.938)	71.52 (9.813)	72.27 (7.919)	0.698	72.84 (8.840)
Male (*n*, %)	92 (73.0)	31 (64.6)	27 (73.0)	0.410	13 (68.4)
*Living status (n, %)*					
Single	30 (23.8)	14 (29.2)	10 (27.0)	0.828	6 (31.6)
Cohabitation (partner/children/other)	96 (76.2)	34 (70.8)	27 (73.0)		13 (68.4)
*Educational level (n, %)*					
Primary school	32 (25.4)	9 (18.8)	12 (32.4)	0.157	5 (26.3)
Upper secondary school	39 (31.0)	15 (31.3)	11 (29.7)		5 (26.3)
University	55 (43.7)	24 (50.0)	14 (37.8)		9 (47.4)
ISI SUM (MD^c^, range)	11.00 (24, 0–24)	15.00 (15, 9–24)	13.00 (13, 8–21)	<0.05	16 (16)
*ISI (n, %)*					
No clinical insomnia 0–7	41 (32.5)	–	–		
Subthreshold insomnia 8–14	37 (29.4)	15 (31.3)	22 (28.2)	<0.05	
Clinical insomnia of Moderate severity 15–21	41 (32.5)	26 (54.2)	15 (19.2)		
Sever clinical insomnia 22–28	7 (5.6)	7 (14.6)	–		
*ISI item 1. Difficulty falling asleep (n, %)*					
None	38 (52.5)	9 (18.8)	6 (16.2)	0.587	
Mild	34 (27.0)	10 (20.8)	9 (24.3)		
Moderate	30 (23.8)	14 (29.2)	13 (35.1)		
Severe	18 (14.3)	9 (18.8)	9 (24.3)		
Very	6 (4.8)	6 (12.5)	–		
*ISI item 2. Difficulty staying asleep (n, %)*					
None	9 (7.1)	2 (4.2)	5 (13.5)	0.126	
Mild	28 (22.2)	2 (4.2)	18 (48.6)		
Moderate	50 (39.7)	20 (41.7)	13 (35.1)		
Severe	30 (23.8)	16 (33.3)	1 (2.7)		
Very	9 (7.1)	8 (16.7)	–		
*ISI item 3. Problem waking up early (n, %)*					
None	23 (18.3)	4 (8.3)	3 (8.1)	0.428	
Mild	33 (26.2)	9 (18.8)	7 (18.9)		
Moderate	40 (31.7)	17 (35.4)	15 (40.5)		
Severe	21 (16.7)	9 (18.8)	12 (32.4)		
Very	9 (7.1)	9 (18.8)	–		
*ISI item 4. How satisfied/dissatisfied are you with your current sleep pattern? (n, %)*					
Very satisfied	9 (7.1)	–	2 (5.4)	<0.001	
Satisfied	25 (19.8)	5 (10.4)	15 (40.5)		
Somewhat	28 (22.2)	34 (70.8)	17 (45.9)		
Dissatisfied	52 (41.3)	9 (18.8)	3 (8.1)		
Very dissatisfied	12 (9.5)	5 (10.4)	–		
**PSQI (*n*, %)**					
*PSQI item 6. How often have you taken medicine to help you sleep?*					
Not during the past month	101 (80.2)	29 (60.4)	32 (86.5)	0.010	
Less than once a week	7 (5.6)	4 (8.3)	2 (5.4)		
Once or twice a week	6 (4.8)	6 (12.5)	3 (8.1)		
Three or more times a week	12 (9.5)	9 (18.8)	–		
*PSQI component 4. Sleep efficiency (n, %)*	*n* = 118^a^	*n* = 43	*n* = 36	0.105	
>85%	35 (27.8)	9 (18.8)	5 (13.5)		
75–84%	34 (27.0)	5 (10.4)	14 (37.8)		
65–74%	14 (11.1)	6 (12.5)	7 (18.9)		
<65%	35 (27.8)	23 (47.9)	10 (27.0)		

^a^ Eight missing values due to un‐valid reported data.

^b^ *p*‐value significant at the 0.05 level.

^c^ Median.

### 4.1 Correlations between sleep variables and the physical and psychological characteristics

Associations regarding insomnia, physical and psychological characteristics (i.e. ISI summary score, ISI‐items 1–4 and PSQI components 4 and 6) are presented in Table 4. Positive significant correlations were found between the somatic symptom severity and insomnia variables (*p* = 0.001, *R*_*s*_ = 0.309–0.597) suggesting that worse somatic symptom severity is related to sleep difficulties. Moreover, the EQ5D had a negative significant association with all the sleep variables, indicating that lower health‐rated quality of life is associated with a worse self‐rated sleep situation. The SF‐12 PCS and MCS had a negative significant association with six of seven insomnia variables. The SF‐12 MCS had a negative significant association with the ISI summary score. The PHQ‐9 had a positive significant correlation with the insomnia variables (*p* = 0.001, *R*_*s*_ = 0.355–0.709), indicating that a higher number of self‐rated depressive symptoms are associated with a higher ISI score, difficulty falling or staying asleep or waking up to early, worse sleep pattern satisfaction, lower sleep efficiency or frequent use of sleep medication. The CAQ item 4 regarding ‘chest pain/discomfort wakes me up at night’ had a positive significant correlation to the ISI summary score, difficulty falling or staying asleep, waking up early, sleep pattern satisfaction and sleep efficiency. The CAQ summary score regarding heart‐focused attention had a positive significant association with all sleep variables except the frequent use of medication to improve sleep (Table 4). The MCS summary score had a negative significant correlation with all sleep variables, indicating that a lower sense of mastery is related to self‐rated sleep difficulties.

**TABLE 4:** Correlations (Spearman's rho) between sleep and the physical and psychological characteristics of patients with CVD interested in participating in the internet‐based cognitive behavioural therapy for insomnia (*n* = 126).

Variable	ISI sum	ISI item 1	ISI item 2	ISI item 3	ISI item 4	PSQI component 4	PSQI item 6
*Demographics*							
Age	−0.029	0.120	0.092	−0.145	−0.060	0.188^a^	0.131
Living status	−0.206^a^	−0.218^a^	−0.148	−0.195^a^	−0.142	−0.108	−0.147
Educational level	−0.003	−0.194^a^	0.005	0.037	0.030	−0.092	−0.184^a^
Myocardial infarction	−0.032	−0.022	−0.042	−0.030	0.009	−0.018	0.066
*Physical characteristics*							
Angina pectoris	0.037	0.088	−0.081	0.116	0.019	−0.115	−0.168
Heart failure	−0.072	0.018	−0.035	−0.058	−0.115	0.076	−0.034
Atrial fibrillation	−0.012	0.004	0.016	−0.107	−0.106	0.177	0.031
Arrhythmia	0.156	−0.055	0.106	0.179^a^	0.219^a^	−0.025	0.095
High blood pressure	−0.088	−0.039	−0.042	−0.156	−0.113	−0.157	−0.050
Lipid disorder	0.237^b^	0.238^b^	0.136	0.127	0.142	0.014	0.200^a^
BMI	0.005	−0.058	−0.018	0.090	−0.078	−0.058	−0.064
Number of medications	0.075	0.153	0.084	−0.033	−0.045	0.067	0.117
SF‐12 PCS	−0.410^b^	−0.381^b^	−0.262^b^	−0.131	−0.295^b^	−0.428^b^	−0.360^b^
SF‐12 MCS	−0.440^b^	−0.318^b^	−0.235^b^	−0.331^b^	−0.376^b^	−0.217^a^	−0.140
PHQ‐15 summary score	0.597^b^	0.397^b^	0.423^b^	0.309^b^	0.431^b^	0.387^b^	0.339^b^
PHQ‐15 Severity level of somatic symptoms	0.572^b^	0.384^b^	0.409^b^	0.289^b^	0.410^b^	0.390^b^	0.330^b^
EQ‐5D summary score	−0.465^b^	−0.380^b^	−0.292^b^	−0.193^a^	−0.359^b^	−0.363^b^	−0.265^b^
EQ‐5D severity level	−0.437^b^	−0.336^b^	−0.264^b^	−0.183^a^	−0.327^b^	−0.352^b^	−0.306^b^
*Psychological characteristics*							
PHQ‐9 summary score	0.709^b^	0.496^b^	0.428^b^	0.450^b^	0.644^b^	0.372^b^	0.355^b^
CAQ Heart‐focused fear and concerns	0.276^b^	0.221^a^	0.164	0.134	0.244^b^	0.037	0.060
*CAQ item 3*	0.339^b^	0.130	0.228^a^	0.240^b^	0.270^b^	0.103	0.065
*CAQ item 4*	0.401^b^	0.338^b^	0.321^b^	0.220^a^	0.247^b^	0.253^b^	0.163
CAQ Heart‐focused avoidance	0.180^a^	0.198^a^	0.089	−0.009	0.112	0.222^a^	0.069
CAQ Heart‐focused attention	0.335^b^	0.193^a^	0.277^b^	0.222^a^	0.299^b^	0.191^a^	0.156
MSC summary score	−0.425^b^	−0.290^b^	−0.246^b^	−0.262^b^	−0.324^b^	−0.355^b^	−0.225^a^
DS‐14 NA/SI score	0.218^a^	0.203^a^	0.074	0.216^a^	0.138	0.117	0.048

^a^ Correlation is significant at the 0.05 level (2‐tailed).

^b^ Correlation is significant at the 0.01 level (2‐tailed).

### 4.2 Characteristic differences of those eligible for and excluded from the intervention

There were no statistically significant differences between the groups eligible for randomisation (*n* = 48) and excluded (*n* = 37) regarding age, gender, living status, educational levels (Table 3), other comorbidities, number of medications or BMI (Table 5). Regarding CVD diagnosis, atrial fibrillation was significantly more common among the excluded than the eligible participants (*x* ^*2*^ = 4.942, *p* = 0.026). The eligible participants reported significantly worse sleep pattern satisfaction (*z* = −3.499, *p* = <0.001) and they were also more likely to take medicine several times a week to improve sleep (*z* = −2.591, *p* = 0.010) than the excluded (Table 3). The eligible participants also had worse somatic symptom severity (*z* = −2.295 *p* = 0.022) and were more likely to report a medium‐ to high‐level of symptom severity than the excluded participants. The health‐rated quality of life levels was statistically non‐significant between the groups, even if the results indicate that the eligible tended to report a lower self‐perceived health state than the excluded participants (*z* = −1.901 *p* = 0.057; Table 5). A significantly higher number of depressive symptoms (*z* = −2.984, *p* = 0.007) were found among the eligible participants, who reported a higher prevalence of mild (35%) and moderate (15%) depressive symptoms than the excluded participants (Table 6).

**TABLE 5:** Physical characteristics of patients with CVD interested in participating in I‐CBTI (*n* = 126).

Variables	Total	Eligible for I‐CBTI ISI 8–28	Excluded ISI 8–28	*P‐value* ^a^	Interview participants
*n*	126	48	37		19
*CVD (n, %)*					
Myocardial infarction	68 (46.0)	26 (54.2)	11 (29.7)	0.135	7 (36.8)
Angina pectoris	36 (28.6)	14 (29.2)	5 (13.5)	0.955	7 (36.8)
Heart failure	13 (10.3)	3 (6.3)	6 (16.2)		1 (5.3)
Atrial fibrillation	37 (29.4)	10 (20.8)	16 (43.2)	0.026	5 (26.3)
Arrhythmia	19 (15.1)	9 (18.8)	6 (16.2)	0.761	
*Other comorbidities (n %)*					
High blood pressure	41 (32.5)	14 (29.2)	13 (35.1)	0.558	
Lipid disorder	46 (36.5)	21 (43.8)	13 (35.1)	0.422	
Diabetes	17 (13.5)	7 (14.6)	5 (13.5)	0.888	
*n*	*124*	*48*	*37*		
Number of medications (MD^b^, range)	4 (12, 0–12)	5 (12, 0–12)	5 (12, 0–12)	0.714	
*n*	126	48	37		
Body mass index (MD, range)	26.42 (29.16–44)	25.95 (24)	26.51 (22)	0.491	
PHQ‐15 (MD, range)	7.00 (20, 0–20)	10.00 (18, 2–20)	4.00 (12, 2–14)	0.022	
*Somatic symptom severity level (n, %)*					
Minimal	35 (27.8)	5 (10.4)	9 (24.3)	0.015	
Low	51 (40.5)	18 (37.5)	16 (43.2)		
Medium	33 (26.2)	18 (37.5)	12 (32.4)		
High	7 (5.6)	7 (14.6)	–		
*SF‐12 (MD, range)*					
SF‐12 PCS	44.97 (47, 15–61)	39.54 (40, 22–61)	44.22 (45, 15–60)	0.142	
SF‐12 MCS	53.80 (49, 19–68)	50.89 (46, 21–68)	49.82 (44, 19–63)	0.842	
EQ‐5D (MD, range)	75.00 (75, 25–100)	70.00 (74, 25–99)	75.00 (75, 25–100)	0.132	
*EQ‐5D Visual analogue health scale 0–100 (n, %)*					
0–50	23 (18.3)	14 (29.2)	6 (16.2)	0.057	
51–75	51 (40.5)	24 (50.0)	17 (45.9)		
76–100	52 (41.3)	10 (20.8)	14 (37.8)		

Abbreviations: CVD, cardiovascular disease; I‐CBTI, internet‐based cognitive behavioural therapy for insomnia.

^a^ *p*‐value significant at the 0.05 level.

^b^ Median.

**TABLE 6:** Psychological characteristics of patients with cardiovascular disease interested in participating in internet‐based cognitive behavioural therapy for insomnia (I‐CBTI; *n* = 126).

Variables	Total	Eligible for I‐CBTI ISI 8–28	Excluded ISI 8–28	*p*‐value^a^
*n*	126	48	37	
PHQ‐9 summary (MD^b^, range)	3.00 (19, 0–19)	6.00 (18, 1–19)	3.00 (17, 0–17)	<0.05
*PHQ‐9 levels (n, %)*				
Minimal 0–4	80 (63.5)	21 (43.8)	25 (67.6)	<0.05
Mild 5–9	33 (26.2)	17 (35.4)	9 (24.3)	
Moderate 10–14	7 (5.6)	7 (14.6)	0	
Moderately severe 15–19	6 (4.8)	3 (6.3)	3 (8.1)	
CAQ (MD, range)				
CAQ‐ fear/concerns	1.06 (2.75)	1.125 (2.75)	1.125 (2.5)	0.943
CAQ‐ avoidance	1.00 (3.6)	1 (3.40)	1 (3.6)	0.650
CAQ‐ heart‐focused attention	0.8 (2.6)	0.8 (2.4)	1 (2.6)	0.668
*CAQ item 3. My racing heart wakes me up at night (n, %)*				
Never	87 (69.0)	28 (58.3)	24 (64.9)	0.518
Rarely	17 (13.5)	8 (16.7)	6 (16.2)	
Sometimes	20 (15.9)	11 (22.9)	6 (16.2)	
Often	2 (1.6	1 (2.1)	1 (2.7)	
*CAQ item 4. Chest pain/discomfort wakes me up at night (n, %)*				
Never	80 (63.5)	27 (56.3)	19 (51.4)	0.871
Rarely	30 (23.8)	11 (22.9)	12 (32.4)	
Sometimes	16 (12.7)	10 (20.8)	6 (16.2)	
*CAQ item 5. I take it easy as much as possible (n, %)*				
Never	19 (15.1)	5 (10.4)	5 (13.5)	0.504
Rarely	39 (31.0)	12 (25.0)	13 (35.1)	
Sometimes	42 (33.3)	20 (41.7)	10 (27.0)	
Often	19 (15.1)	9 (18.8)	6 (16.2)	
Always	7 (5.6)	2 (4.2)	3 (8.1)	
MCS summary (MD, range)	23.00 (17, 11–28)	22 (16, 11–27)	21.00 (14, 14–28)	0.908
*MCS item 4. I can do just anything I really set my mind to (n, %)*				
Strongly disagree	19 (15.1)	4 (8.3)	8 (21.6)	0.182
Agree as much as disagree	23 (18.3)	10 (20.8)	9 (24.3)	
Agree	57 (45.2)	25 (52.1)	13 (35.1)	
Strongly agree	27 (21.4)	9 (18.8)	7 (18.9)	
DS‐14 Type D personality trait (*n*, %)	31 (24.6)	14 (29.2)	11 (29.7)	0.995

^a^ *p*‐value significant at the 0.05 level.

^b^ Median.

### 4.3 The three personae's motives and expectations for participating in an I‐CBTI study

*The pragmatist* is a curious and optimistic persona that has trouble staying asleep and has lived with poor sleep for a long time. *The pragmatist* wishes to have coherent sleep and wants to have an explanation for why the poor sleep situation has occurred. Another motive to participate is that *the pragmatist* believes that the situation cannot get any worse and has nothing to lose by participating and is ready to do something about the sleep situation. There is no fear about trying new and untested treatment methods; however, *the pragmatist* is cautious about taking medical substances and prefers non‐pharmacological treatment options. Moreover, *the pragmatist* is interested in receiving treatment via the internet and already knows that CBT is an effective treatment for other psychological conditions and, therefore, expects the I‐CBTI to be able to improve sleep.

*‘I have the expectations that my sleep situation will be manageable and that I will learn more about cognitive behavioural therapy; that will be exciting as well’*.

*The pragmatist* considers the participation to be helpful for him or herself and others and is confident that the participation will lead to something positive, but also declares that there is no quick fix or any magical solutions that are able to improve the sleep situation.

*The philosopher* is a problem‐solving persona with daytime fatigue due to trouble falling asleep or staying asleep. The motive for participating is to find specific tools to improve sleep and the ability to experience a full night's sleep. *The philosopher* wishes to gain knowledge about the characteristics of normal sleep and to change the negative aspects of lifestyle regarding sleep behaviour. The motives for participating are to use the retrieved sleeping tools when having difficulty falling asleep when having troubling thoughts at night or having difficulty staying asleep.

*‘I think maybe that I could change my lifestyle, that there is something wrong with it, that maybe I go to bed too late or get up too late or that I could do something else’*.

*The philosophe*r expects to learn about I‐CBTI effectiveness but has realistic expectations that some sleep habits might remain, such as recurrent awakenings. There is also a worry that the present comorbid conditions may be the cause of the sleep disturbances and might influence the ability to improve sleep. Nevertheless, *the philosopher's* expectations are that the participation will reveal new knowledge that could help with the poor sleep situation.

*The philanthropist* is an altruistic persona with trouble falling or staying asleep and has great confidence in research, and expects the participation in the I‐CBTI study to be useful and meaningful for others in the present or the future. *The philanthropist* does not describe the sleeping problem as the primary motive to participate even though there is a wish to improve the sleep situation. *The philanthropist* has no clear ideas on how this will be done and hopes participation will lead to some general sleep advice, even though there is a belief that the existing problems with poor sleep, heart disease and other current health conditions may be difficult to solve. However, *the philanthropist* is curious about what the participation will lead to, without having any expectations that it will improve sleep or the heart condition.

*‘I have no expectations at all, I do not. I was just thinking, okay maybe my experience can help someone else’*.

*The philanthropist's* motive to participate is partly influenced by a family member who thought that participation would be beneficial, and there is no demand for participation to solve all problems, but *the philanthropist* hopes that it might lead to some help with the sleep situation for him or herself and others.

## 5 DISCUSSION

This study shows that primary care patients with CVD and insomnia, interested in participating in an I‐CBTI study, had a mean age above 70, were mainly male, cohabited with another person and had a university education. It was evident that the participants´ health‐related quality of life, somatic symptoms, depressive symptoms and cardiac anxiety scores were significantly associated with several insomnia variables. Moreover, motives to participate and expectations of the *pragmatist, philosopher* and *philanthropist* show that there are behavioural features to consider when evaluating a patient's motive to adapt and adhere to such treatment.

We found that 30% of the participants who were randomised to the I‐CBTI had assessed themselves as having subthreshold insomnia (i.e., ISI score 8–14). This could be a problem since lower insomnia severity has proven to be related to poorer treatment adherence in I‐CBTI and early dropout (Yeung et al., 2015). Therefore, lower insomnia severity might possibly explain why the persona *the philanthropist* lacked clear goals to improve their sleep problem and therefore be less motivated to adhere to I‐CBTI. Another issue is that insomnia can be present with other types of sleeping disorders, such as obstructive sleep apnoea, circadian sleep–wake disorders or parasomnias (Bjorvatn et al., 2021). Therefore, it is important to set an optimal ISI cut‐off to prevent false positive responses and determine a clinical insomnia diagnosis both by validated insomnia questionnaires in combination with clinical examinations (i.e. including inquiring whether the patient identifies themselves as a person with insomnia) to ensure that the patients have a type of insomnia that is suitable for an I‐CBTI.

Depressive symptoms and somatic symptom severity were significantly correlated with the insomnia variables in the current study, and those who were eligible for the I‐CBTI study reported worse somatic symptom severity and higher depressive symptoms than the excluded participants. Depression is a well‐known comorbidity of CVD and/or insomnia (Javaheri & Redline, 2017; Johansson et al., 2016), and in the current study, 90% of the participants were assessed as having minimal to mild depressive symptoms, which might be a factor behind treatment dropouts (Hebert et al., 2010). However, I‐CBTI has also been shown to improve depressive symptoms and fatigue (Hagatun et al., 2018), and minimal to mild depressive symptoms should therefore not mean patients with CVD and insomnia are unsuitable for I‐CBTI. Also*, the philosopher* and *the philanthropist* were doubtful that the insomnia could be improved given the existing comorbid conditions, and *the philosopher* believed the comorbidity to be the actual cause of the insomnia. The insomnia variables in the current study were significantly associated with heart‐focused attention, and the participants*'* reports on whether chest pain/discomfort awakened them at night, could imply that cardiac symptoms influence the sleep situation and similar experiences are described by patients with CVD and insomnia in a previous study (Siebmanns et al., 2020). The physical and psychological associations with insomnia revealed in the statistical analyses, and *the philosopher's* and *the philanthropist's* thoughts on how the comorbid conditions might influence the ability to improve sleep, indicate that it is important to include information on physical (somatic and cardiac symptoms) and psychological consequences (depression and cardiac anxiety) of CVD and insomnia to improve adherence for I‐CBTI. Also, further studies are needed to investigate depressive and cardiac symptoms among patients with CVD and how they influence treatment adherence.

Eighty percent of the participants in the current study reported they had not used medication to help with sleep, which could mean that the participants were searching for other insomnia treatment options, such as I‐CBTI. This also reflects *the pragmatist's* desire to use non‐pharmacological treatment methods and *the philosopher's* search for knowledge about sleep tools to change negative aspects of lifestyle regarding sleep behaviour. Also, *the pragmatist* and *the philosopher* both have specific motives and expectations to find a solution for their insomnia, which relates to the 45% who, via the Mastery Scale, agreed they could do just about anything they really set their mind to. According to the trans‐theoretical model of health behaviour change (Prochaska et al., 1993), the process of change also depends on time. This means that when a person is motivated and ready to act on a behavioural change, they may intend to implement this change in the next 6 months or in the immediate future. Moreover, the intention to act is also connected to a person's awareness of the pros, which should be greater than the cons for a person to consider behavioural change (Prochaska et al., 1993). *The pragmatist* and *the philosopher* might see benefits to the I‐CBTI and be ready to take action for behavioural change in the near future and therefore may be suitable for an action‐oriented treatment (Prochaska & Velicer, 1997). *The philanthropist*, however, hopes to receive general sleep advice and has a low outcome expectancy. This might be explained by a low self‐efficacy, which is an important aspect of behavioural change, that operates in relation to a positive outcome expectancy (Schwarzer, 2008). Low treatment expectancy can also be explained by type D personality traits, which were reported among 25% of the participants. Patients with CVD and type D‐personality rarely engage in health‐promoting behaviours and are more likely to perceive their illness as severe and less manageable, which may influence their commitment to health‐promoting behaviours (Cao et al., 2016). Most of the participants in the current study cohabited with another person (76%), and social support is suggested to decrease stress among patients with type D‐personality and to enhance intervention effects (Cao et al., 2016). Self‐efficacy has been shown to be an important factor in relation to social support as it has been suggested to influence adherence behaviour among patients with heart failure (Maeda et al., 2013). Future research should therefore focus on the quality of social support, the influence of type D personality traits and depression symptoms on adherence to I‐CBTI, as well as the user experience of such an intervention.

### 5.1 Limitations

The quantitative sample was relatively small, and the CVD diagnoses were unevenly distributed. Moreover, there were more men than women participants, which in part can be explained by the higher prevalence of a CVD diagnosis among men. Another limitation was the use solely of self‐assessed measurements; however, a strength is that the insomnia diagnosis was defined by a physician with extensive experience in clinical neurophysiology and the CVD diagnosis was determined by the healthcare service. Moreover, the validity of the persona model has not been widely studied, the qualitative data includes several dimensions to consider during the analysis and it is difficult to verify the accuracy of the created personas. Even so, the qualitative analysis was conducted by the first author with guidance from the co‐authors, who had considerable experience in qualitative research, which strengthens the trustworthiness of the results.

## 6 CONCLUSION

This study shows that primary care patients with CVD interested in participating in an I‐CBTI study have physical (somatic and cardiac symptoms) and psychological characteristics (depression and cardiac anxiety) related to insomnia. Moreover, their motives and expectations are that the I‐CBTI will improve their insomnia, although they have some concerns that the comorbidities might influence their ability to implement the necessary acquired behavioural change. Nurses should assess and monitor physical and psychological characteristics related to insomnia, as well as the motives and expectations to implement the necessary behavioural change, these aspects should be evaluated with valid methods in the eligibility procedure for I‐CBTI to improve the adherence. Moreover, future research should investigate the experiences of using I‐CBTI for patients with CVD and insomnia.

## AUTHOR CONTRIBUTIONS

SS designed the study, collected, analysed and interpreted the patient data with support from LJ, JS, MG, and AB. SS created the initial draft of the manuscript. All the authors contributed constructive criticism during the process and approved the final version of the manuscript.

## FUNDING INFORMATION

Not applicable.

## CONFLICT OF INTEREST STATEMENT

No conflicts of interest.

## ETHICS STATEMENT

All participants gave their informed written consent to participate in the I‐CBTI study. The research project was approved by the Regional Ethical Council, Linköping University (Dnr 2015/258–31, 2017/378–32).

## CONSENT FOR PUBLICATION

Not applicable.

## Supporting information


Appendix S1.

## Data Availability

The datasets generated and/or analysed during the current study are not publicly available due to ethical restrictions but are available from the corresponding author on reasonable request.
