# Supplementary material for: Primary care patients with cardiovascular disease eligible for nurse‐led internet‐based cognitive behavioural therapy for insomnia: Characteristics and motives for participation
Source: Nurs Open. 2023 Mar 17;10(7):4676–89. doi: 10.1002/nop2.1717 (PMC10277389; doi:10.1002/nop2.1717)
Supplement: Supplementary file 1 — Appendix S1. [file NOP2-10-4676-s001.docx]

**QUANtitative data collection**

Baseline intervention measurements (web-based questionnaires)

**Appendix 1**. A visual description of the mixed-method design of the study

**QUANtitative analysis**

Descriptive statistic frequencies, group comparison and correlations

**Integration of QUALitative and QUANtitative results**

Discussion, implications
and future research

**QUALitative analysis**

Personas model

**QUALitative data collection and QUAN and QUAL integration**

Audiotaped face-to-face interviews
(participants selected based on measurement scores and
clinical examination outcomes)
